# Investigation of the 0.4C-35Cr-45Ni-Nb Alloy after Service in High-Temperature Steam and Hydrocarbons Environment

**DOI:** 10.3390/ma14206139

**Published:** 2021-10-15

**Authors:** Juliusz Orlikowski, Michał Szociński, Janusz Zygmuntowicz, Gabriel Gajewski, Wojciech Filipkowski, Kazimierz Darowicki

**Affiliations:** 1 Department of Electrochemistry, Corrosion and Materials Engineering, Chemical Faculty, Gdańsk University of Technology, G. Narutowicza Str. 11/12, 80-233 Gdańsk, Poland; juliuszo@pg.edu.pl (J.O.); kazdarow@pg.edu.pl (K.D.); 2 PKN Orlen S.A., Chemików Str. 7, 09-411 Płock, Poland; janusz.zygmuntowicz@orlen.pl (J.Z.); gabriel.gajewski@orlen.pl (G.G.); wojciech.filipkowski@orlen.pl (W.F.)

**Keywords:** chromium-nickel alloy, pyrolytic furnace, high-temperature degradation, hydriding, mechanical properties deterioration

## Abstract

The paper presents the results of investigation of the 0.4C-35Cr-45Ni-Nb alloy, which operated in the cracked hydrocarbon feeds and dilution steam at 1125 °C. The material originated from the pyrolytic furnace coil tubes, of which internal walls were in contact with the aforementioned medium, whereas the external walls were in contact with the flue gases. The examination included metallographic and mechanical tests on the material after service exposure, the results of which were compared with the ones obtained for the as-received non-exposed specimens. The metallographic tests revealed changes in the alloy’s structure manifested by formation of significant amount of the carbides due to carburization of the alloy from the steam and cracked hydrocarbon feeds side. The central and external parts of the alloy samples (having no contact with the process medium) underwent substantial degradation but within a relatively narrow zone of the material. The investigations of hydrogen and methane content in the alloy showed a high amount of these gases, resulting from high-temperature corrosion in steam environment. The mechanical tests demonstrated clear shortening of the plastic deformation range of the alloy due to penetration of the gases and formation of the carbides inside the material’s structure. A low level of corrosion and no creep mechanism were detected.

## 1. Introduction

The 0.4C-35Cr-45Ni-Nb alloy belongs to relatively new construction materials employed in the design of the furnaces operating at high temperatures in the petrochemical industry. The niobium content provides high resistance to creep at temperatures up to 1150 °C [1]. Moreover, the alloy is characterized by high resistance to carburization and metal dusting in hydrocarbon environments [2]. It is applied typically in the petrochemical industry for construction of the furnaces as well as in the petroleum industry, mainly in reforming installations. The investigated alloy samples originated from the coil tubes of the pyrolytic furnace, where olefins are obtained from the cracked hydrocarbon feeds. The feed for the furnace is obtained directly from crude oil distillation. Dilution steam is added to the feed, and it constitutes up to 65% wt. of the entire technological stream. The maximum stream temperature is maintained at 805–866 °C. The technological process is accompanied by coke formation as a result of dehydrogenation reaction [2]. The coke is removed by periodical decoking using a steam-air mixture [2]. The coal formed on the internal walls of the furnace’s coil tubes favors carburization and metal dusting processes due to high service temperatures [3]. Additionally, the interaction between cracked hydrocarbon feeds and steam can yield erosion processes, sulfidation and polythionic acid stress corrosion cracking. High temperature can result in numerous degradation processes involving structural changes of the material such as creep, reheat cracking and sigma phase embrittlement [3]. Decarburization and fuel ash corrosion are the processes that can occur from the heated side of the coil tubes [3]. Selection of the construction materials for the furnace’s coil tubes is accomplished at a designed stage, taking into account the coil tube wall temperature, pressure inside the installation, corrosivity of the medium inside the coil tubes as well as corrosion aggressiveness of the environment outside the coil tubes (flames of combusted gas) [4]. Actual service conditions often differ from the ones predicted upon design, which contributes to high cost of premature replacement of the furnace’s parts [5]. Mechanical and metallographic tests as well as the analysis of the technological medium composition are useful in failure prevention and limitation of the potential repair cost. The aim of this paper is the corrosion risk assessment of the pyrolytic furnace’s coil tubes after their scheduled service period and the comparison of the results with the available literature data.

## 2. Materials and Methods

The investigations were conducted on the construction material of the coil tubes of a furnace, in which olefins production occurs. The tests were conducted on both service exposed and as-received, non-exposed reference samples. Table 1 presents chemical composition of the alloy (Sandvik AB, Sandviken, Sweden) used for construction of the furnace’s coil tubes.

**Figure 1:**
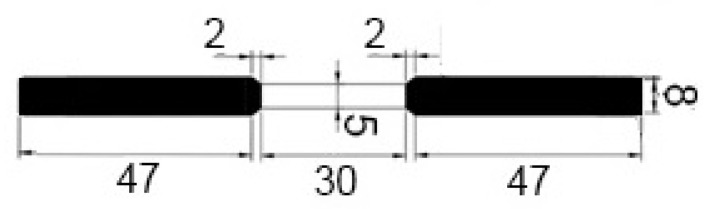
Scheme of the samples employed in the mechanical tests. (All dimensions are expressed in millimeters.)

The furnace had been operating for 5 years when the coil tubes were replaced with the new ones following a procedure defining the maximum admissible service period. The corrosion risk assessment of the furnace’s coil tubes engulfed the following:

- Metallographic investigations were carried out using the metallographic microscope Nikon MA-200 (Nikon Corporation, Tokyo, Japan). The samples were prepared by grinding with abrasive paper of gradation up to 4000 (average particle diameter 2.5 μm) and then by polishing with diamond paste of gradation down to 1 μm. The etching involved a freshly prepared mixture of 10 cm^3^ of 65% nitric acid (HNO_3_), 20 cm^3^ of 40% hydrofluoric acid (HF) and 30 cm^3^ of glycerin (C_3_H_5_(OH)_3_). All solutions were prepared with ACS grade purity reagents from Sigma-Aldrich.
- Scanning electron microscopy (SEM) measurements were performed with the S-3400 N microscope manufactured by Hitachi (Schaumburg, IL, USA). The micrographs were taken in the secondary electron mode for 20 kV accelerating voltage. The imaging involved the backscattered electron (BSE) mode. The microscope was equipped with an EDX detector by ThermoFisher Scientific (Waltham, MA, USA).
- Atomic force microscopy (AFM) and scanning Kelvin probe force microscopy (SKPFM) measurements employed the NTEGRA Aura microscope by NT-MDT (Moscow, Russia), with the head dedicated to Kelvin probe measurement mode. Commercially available NSG01 tips by NT-MDT were utilized for imaging. Additionally, they were vacuum sublimation coated with gold, the thickness of which was 100 nm. The scan rate was equal to 1 line per second. The operating point during topographic scan constituted half of the amplitude of free oscillations at a resonant frequency of the probe. During potential profile recording, the constant height component was 100 nm in order to minimize the influence of short-range interatomic forces.
- The temperature vacuum extraction method was used for the evaluation of hydrogen and methane trapped inside the material. The alloy samples were shredded into small pieces by a slow rotating lathe. Obtained alloy chips were kept in an ultrasonic bath with distilled water for 5 min and then dried at 1100 °C for 1 h. In the next step, the alloy chips were placed under 800 mbar vacuum and heated up to 810 °C. Evolved hydrogen and methane were measured using the Agilent 6890 gas chromatograph (Santa Clara, CA, USA).
- Mechanical tests were performed using the Zwick Z030 tensile testing machine (ZwickRoell GmbH & Co. KG, Ulm, Germany). Figure 1 illustrates the scheme and dimensions of the samples employed in the mechanical investigations. Stress-strain curves were obtained for three service exposed and three non-exposed samples. The strain increment rate was 1 mm/min.

## 3. Results and Discussion

**Figure 2:**
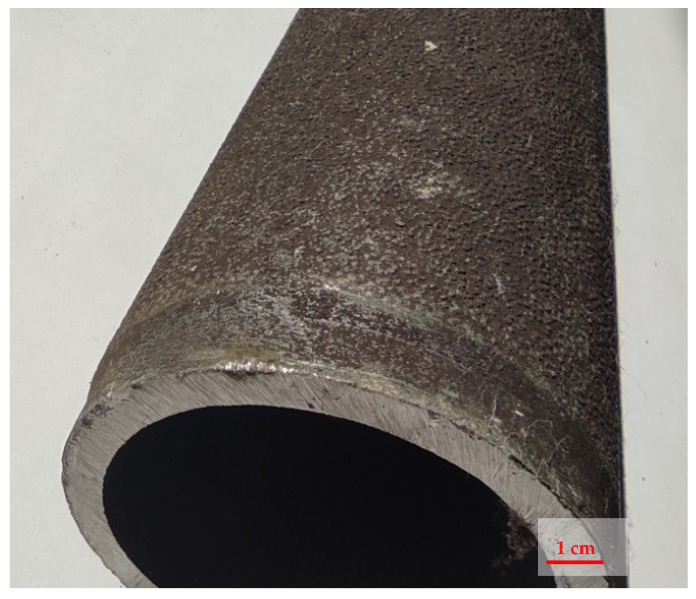
Pyrolytic furnace’s coil tube subjected to investigation.

The investigations were performed on the construction material from the pyrolytic furnace’s coil tube depicted in Figure 2.

The following fragments of the coil tube wall were analyzed:

- Central part, which was subjected to high-temperature impact;
- External part, which was in contact with high temperatures and flue gases originating from combustion of natural gas;
- Internal part, which was subjected to the influence of high temperatures, dilution steam and cracked hydrocarbon feeds.

### 3.1. Results of the Investigation on the Central Part of the Furnace’s Coil Tube

**Figure 3:**
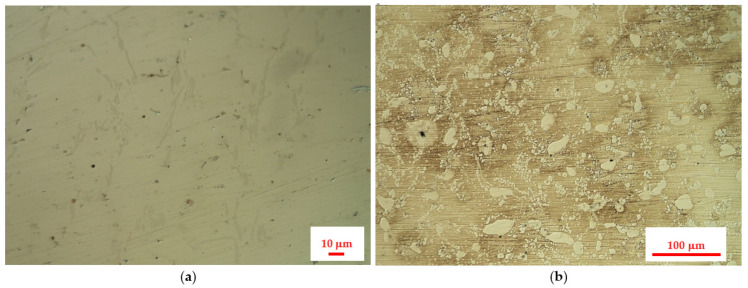
Surface images of the 0.4C-35Cr-45Ni-Nb alloy obtained during metallographic investigations: (**a**) as-received reference sample; (**b**) material after 5 years of service in the furnace.

Figure 3 presents the results of metallographic investigations of the service exposed and as-received reference samples of the 0.4C-35Cr-45Ni-Nb alloy.

**Figure 4:**
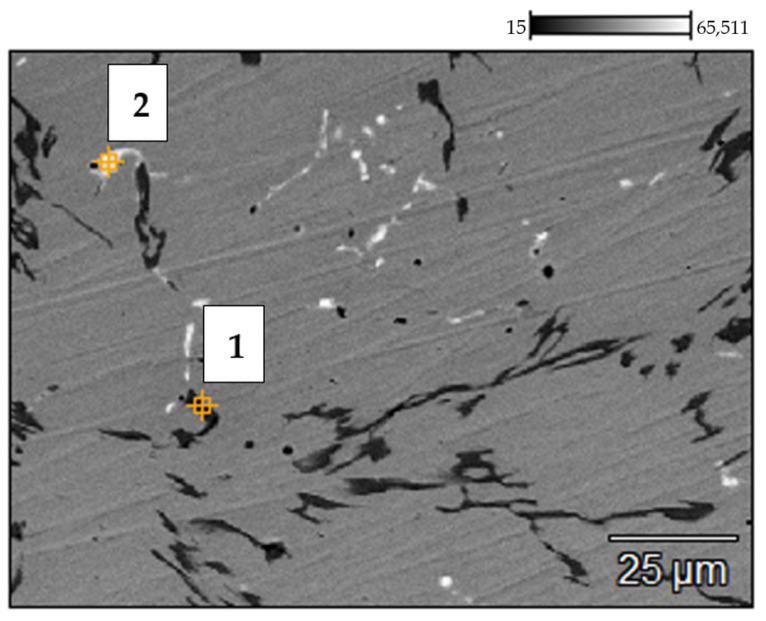
Backscattered electron (BSE) mode scanning electron microscopy (SEM) image of the 0.4C-35Cr-45Ni-Nb alloy—non-exposed reference sample’s surface. Numbers indicate the spots of energy dispersive X-ray (EDX) analysis.

**Figure 5:**
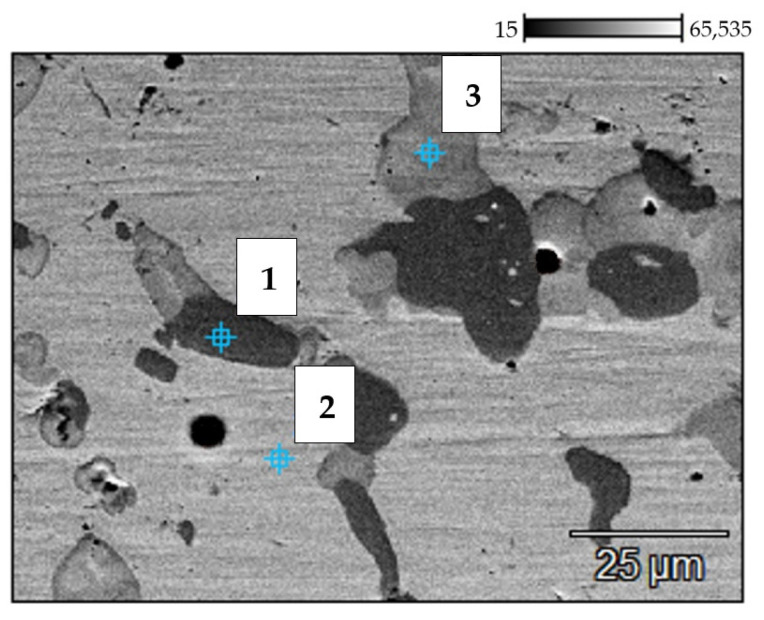
BSE SEM image of the 0.4C-35Cr-45Ni-Nb alloy—service exposed sample’s surface. Numbers indicate the spots of the EDX analysis.

The results of SEM and EDX investigations of the service exposed and as-received reference samples of the 0.4C-35Cr-45Ni-Nb alloy are shown Figure 4 and Figure 5. Table 2 and Table 3 present the elemental composition, expressed as weight percentage, of the reference sample and service exposed sample, respectively.

The results of the investigation of the non-exposed reference sample are in accordance with the literature data [6,7,8]. The metallographic tests revealed carbides present in the form of branched lines (Figure 3a) [9,10]. The SEM image (Figure 4) shows dark structures (spot 1) containing significant amount of chromium and bright structures (spot 3) with higher niobium content with respect to the matrix. Very similar structure and elemental composition of particular phases of the alloy can be found in the paper [8].

In case of the alloy after 5 years of service exposure, one can notice a significant change in the microstructure. There are much more distinct precipitates of the carbides, which occupy a substantial area of the sample (Figure 3b). The change of microstructure results from long-term exposure to the temperature above 805–866 °C and from the influence of the stream inside the coil tubes, which favored carburization [2]. SEM and EDX also reveal chromium-rich phases (spot 1—Figure 5); however, the composition of particular phases differs with respect to the alloys subjected only to an ageing process [2,8]. This fact indicates that the change of microstructure is related to the impact of both aggressive environment and high temperature [11]. No creep process was identified, which suggests a high resistance of the alloy to this phenomenon.

**Figure 6:**
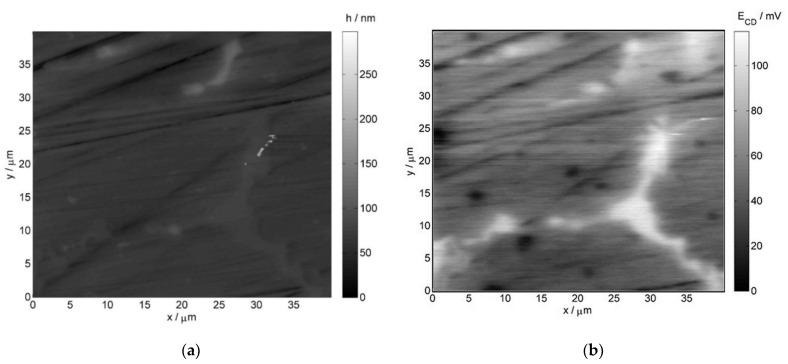
(**a**) AFM and (**b**) SKPFM images of the 0.4C-35Cr-45Ni-Nb alloy—non-exposed reference sample’s surface.

**Figure 7:**
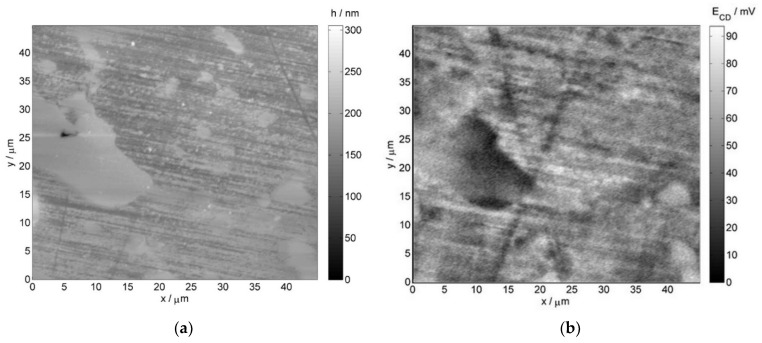
(**a**) AFM and (**b**) SKPFM images of the 0.4C-35Cr-45Ni-Nb alloy—service exposed sample’s surface.

Atomic force microscopy and scanning Kelvin probe force microscopy images were also collected for further verification of the obtained results. Figure 6 illustrates the AFM and SKPFM images for the non-exposed reference sample, whereas Figure 7 depicts the respective images for the service exposed sample. The obtained results reveal structural differences in the material corresponding to the phases of various nobility.

In case of the non-exposed reference sample, the chromium-rich inclusions (more noble) are present on grain boundaries, which is visible on both the AFM image (brighter elevated regions) and SKPFM image (bright regions indicating higher potential as compared to the matrix)—Figure 6. After the service exposure, niobium-rich phases are clearly resolved on the AFM and SKPFM images (Figure 7). They can be identified as brighter regions on the topographical AFM image, whereas on the SKPFM image, the niobium-rich phases are dark regions due to their lower potential with respect to the matrix. These results are consistent with the EDX measurements presented earlier.

### 3.2. Results of the Investigation of the Internal Part of the Furnace’s Coil Tube (the Side of the Technological Stream)

**Figure 8:**
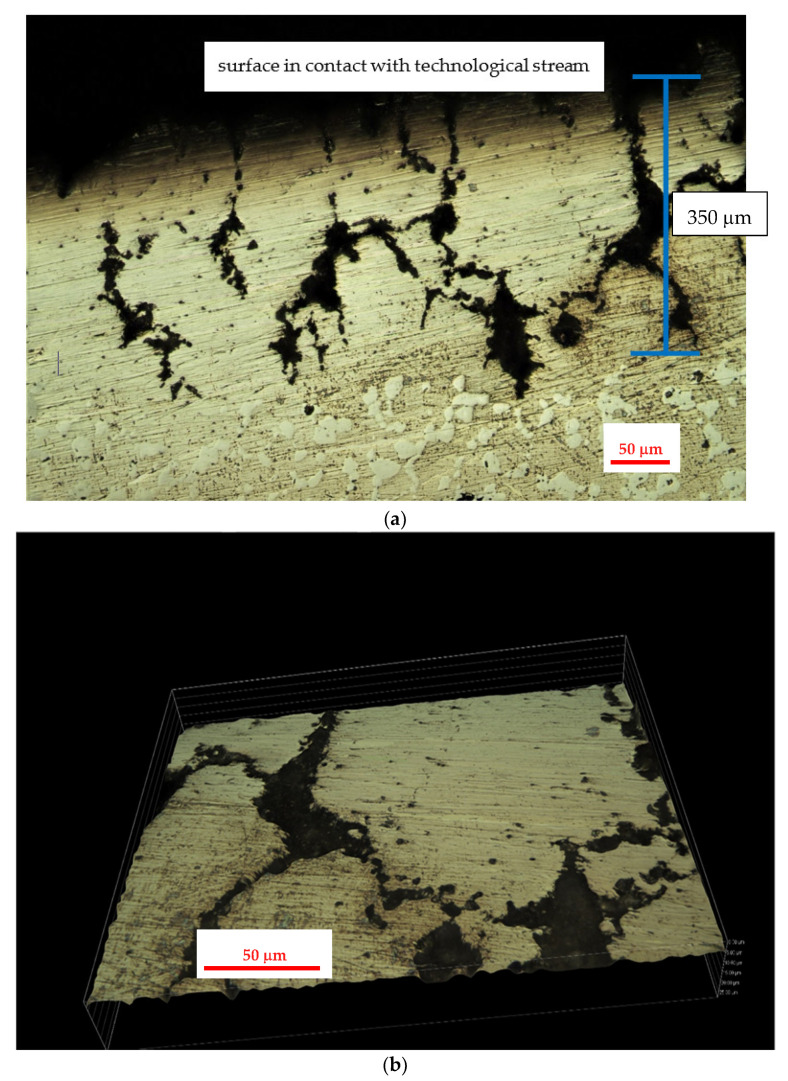
Surface images of the 0.4C-35Cr-45Ni-Nb alloy obtained during metallographic investigations of internal part of service exposed coil tube: (**a**) internal wall neighboring region; (**b**) magnification of a fragment of the region presented in (**a**).

The results of metallographic investigations on the internal part of the service exposed samples of the 0.4C-35Cr-45Ni-Nb alloy are shown in Figure 8.

**Figure 9:**
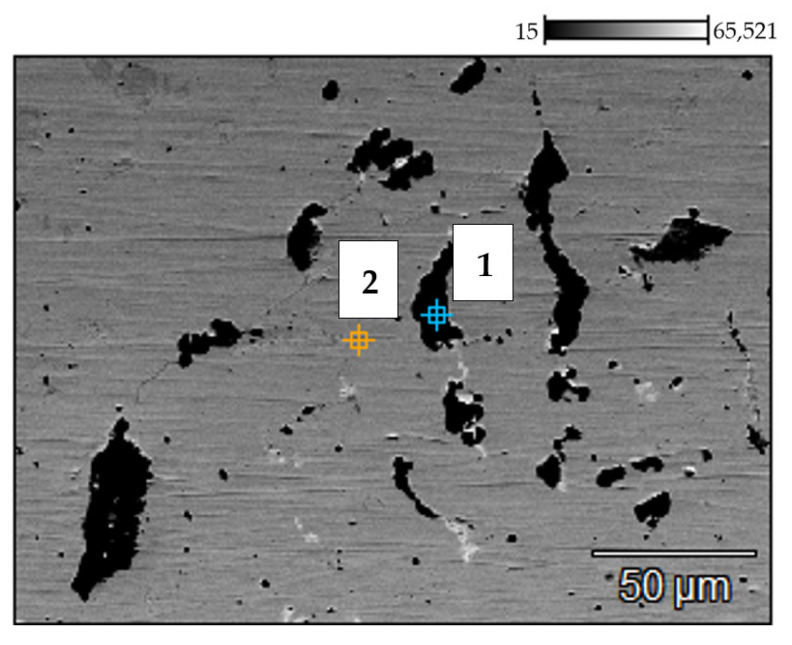
BSE SEM image of the 0.4C-35Cr-45Ni-Nb alloy surface—internal part of the service exposed sample. Numbers indicate the spots of EDX analysis.

Figure 9 presents the SEM image of the internal part of the service exposed sample. The elemental composition of the regions marked 1–2 obtained via the EDX analysis is given in Table 4.

The metallographic investigations show high degradation of the alloy structure in the part of the coil tube. This degradation extends ca. 300 μm from the internal wall to the bulk of the specimen. Visible dark structures are characterized by high amount of silicon and lower content of chromium (spot 1—Figure 9) as compared to the matrix. The presence of a narrow region suffering from dechromization process is also reported in some literature sources [2,12]. Moreover, the degraded zone of the alloy is much more carburized due to the presence of coke, which is formed during thermal degradation of cracked hydrocarbon feeds. Upon service conditions, the internal part of coil tubes can be subjected to metal dusting, although no significant corrosion losses were detected [13,14].

### 3.3. Results of the Investigation of the External Part of the Furnace’s Coil Tube (from the Side in Contact with the Flue Gas)

**Figure 10:**
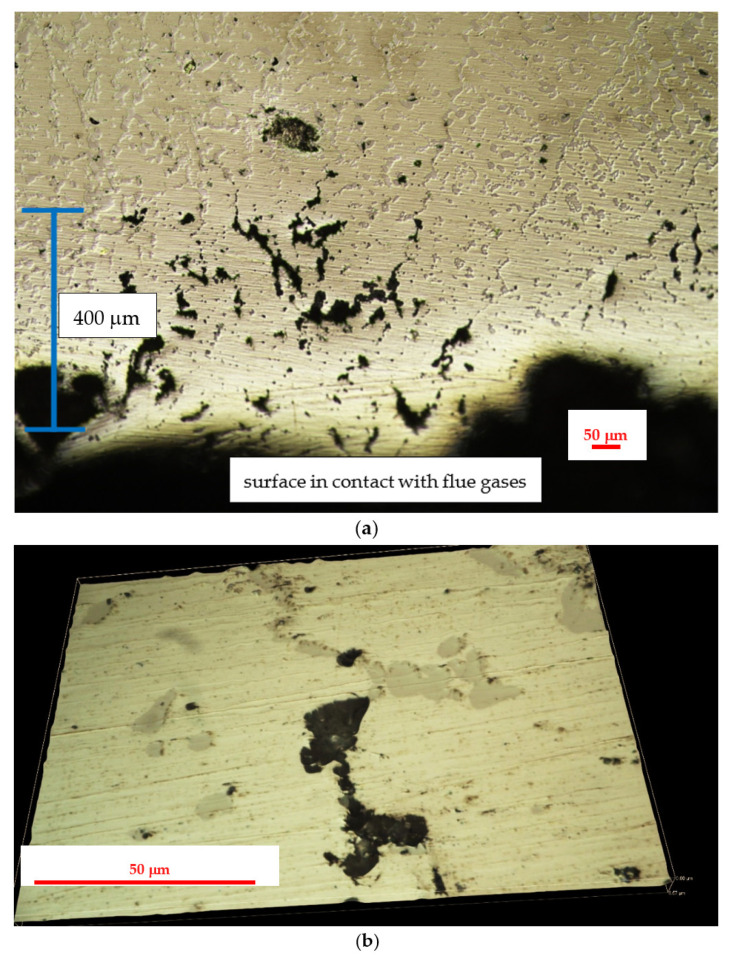
Surface images of the 0.4C-35Cr-45Ni-Nb alloy obtained during metallographic investigations of external part of service exposed coil tube: (**a**) external wall neighboring region; (**b**) magnification of a fragment of the region presented in (**a**).

Figure 10 shows the results of metallographic investigations on the external part of the service exposed samples of the 0.4C-35Cr-45Ni-Nb alloy.

**Figure 11:**
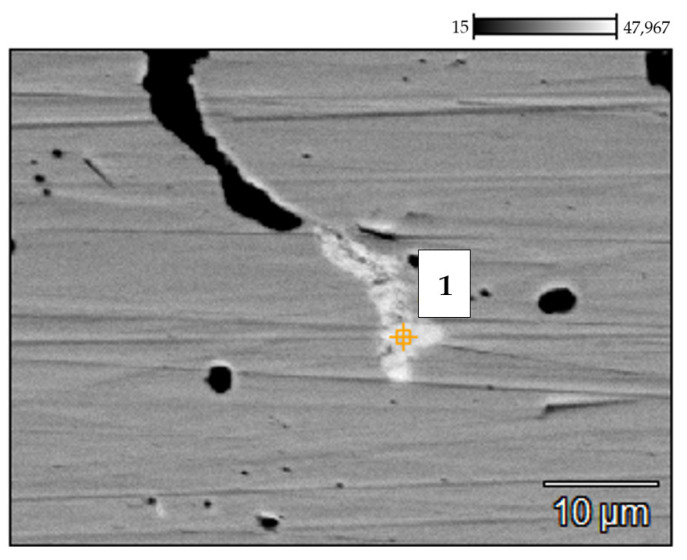
BSE SEM image of the 0.4C-35Cr-45Ni-Nb alloy surface—external part of the service exposed sample. The number indicates the spot of the EDX analysis.

Figure 11 depicts the SEM image of the external part of the service exposed sample. The elemental composition of the region marked 1 obtained via the EDX analysis is given in Table 5.

The external part of the coil tube was in contact with the flue gases used for heating of the furnace. In the analyzed case, the fuel consisted of gas hydrocarbons, mainly methane. Oxygen content in the flue gases stream was low, which indicated no risk of the oxidation mechanism [3]. The gas fuel utilized was of high purity, thus eliminating the mechanisms of fuel ash corrosion and flue gas dew point corrosion [3]. High temperature and low content of carbon compounds in the flue gases resulted in decarburization, which is evident on the metallographic images (Figure 10). It occupies a zone of ca. 200 μm, which corresponds to the region with a limited number of carbides [11]. The entire zone of significant structural degradation covers 400 μm.

### 3.4. Results of the Investigation of the Hydrogen and Methane Content in the Alloy Structure

Table 6 presents the results of the investigation of the hydrogen and methane content in the samples of examined alloy obtained with the temperature vacuum extraction method. The material for the test was collected from the central part of the coil tube specimens.

Obtained results indicate that hydriding and high temperature hydrogen attack processes took place in the investigated the 0.4C-35Cr-45Ni-Nb alloy. Corrosion process occurs in steam and high temperature environment [15]. The effect of corrosion reactions is the evolution of hydrogen, which can be generally described by the following equations [16]:

Fe → Fe^2+^ + 2e^−^ (1)

H_2_O + 2e^−^ → O^2−^ + H_2_ (2)

At high temperatures, the hydrogen produced reacts with the carbon present in the alloy-forming methane. The 0.4C-35Cr-45Ni-Nb alloy is susceptible to such degradation due to its high hardness and significant carbon content.

Regarding corrosion products, at high temperatures, they can include various oxides of iron: FeO, Fe_2_O_3_, and Fe_3_O_4_. Their stability and location within the corrosion products layer depend on material wall temperature. Due to periodical decoking process, which is carried out inside the installation, it is impossible to associate the structure of corrosion product layers with a particular corrosion mechanism in the steam environment.

### 3.5. Results of the Mechanical Tests

**Figure 12:**
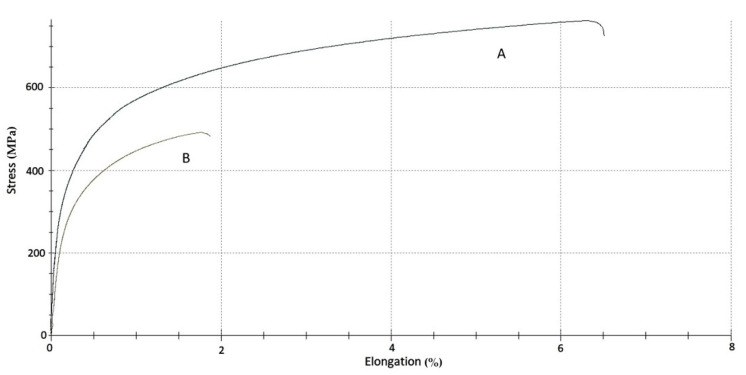
Results of mechanical tests of: (**A**) non-exposed reference sample; (**B**) service exposed sample.

The results of the mechanical investigations on the 0.4C-35Cr-45Ni-Nb alloy are presented in Figure 12 and in Table 7.

Based on the recorded stress-strain curves (Figure 12), the following mechanical parameters were determined and compared: σ for 0.2% plastic deformation, which is the magnitude of stress corresponding to 0.2% of plastic strain, Young’s modulus calculated for the elastic range of the recorded curves, σ_max_, which stands for the maximum tensile stress achieved during the mechanical tests and ε for σ_max_ describing elongation at the maximum load.

The mechanical investigations revealed significant changes of the mechanical properties of the service exposed sample as compared to the reference material. Deterioration of plastic properties of the alloy is evident. The ultimate elongation of the exposed material decreased by more than three times compared to the non-exposed reference specimen. The maximum tensile stress also decreased by ca. 10%. Substantial deterioration of the mechanical properties is probably associated with the process of hydrogen and methane bubbles formation inside the alloy structure due to corrosion processes. Indisputably, it is a dangerous phenomenon (with potential negative influence on impact strength), which calls for more detailed investigation.

## 4. Conclusions

The performed investigations showed that the 0.4C-35Cr-45Ni-Nb alloy underwent structural degradation with a simultaneous lack of significant corrosion loss upon exposure to high temperatures, steam, C6–C8 hydrocarbons, and flue gases. A high degree of deterioration of the mechanical properties was discovered, which can cause susceptibility to vibrations and fatigue. Significant deterioration of the mechanical properties results from a series of degradation and corrosion processes. The degradation processes connected with a change of the microstructure (formation of carbides) and, to some extent, with hydrogen absorption were a decisive factor, which was confirmed by the analytical investigations. The investigations revealed surface and subsurface material degradation caused by the interaction with high temperature and aggressive chemical compounds.

## Figures and Tables

**Table 1:** Chemical composition (weight percentage) of investigated alloy (smelting data).

C (%)	Ni (%)	Cr (%)	Fe (%)	Mn (%)	Si (%)	Nb (%)
0.39	44.92	37.32	13.6	1.11	1.83	0.83

**Table 2:** Elemental composition (weight percentage) of the reference sample obtained by EDX analysis at two spots on the material’s surface (the places marked 1–2 in Figure 4).

No.	C (%)	Si (%)	V (%)	Cr (%)	Mn (%)	Fe (%)	Ni (%)	Nb (%)
1	4.53	0.17	0.24	85.71	0.00	3.73	5.40	0.23
2	4.73	0.65	0.48	37.14	1.25	4.59	5.77	45.39

**Table 3:** Elemental composition (weight percentage) of the service exposed sample obtained by EDX analysis at three spots on the material’s surface (the places marked 1–3 in Figure 5).

No.	C (%)	Si (%)	V (%)	Cr (%)	Mn (%)	Fe (%)	Ni (%)	Nb (%)
1	5.16	0.20	0.00	87.62	0.00	2.32	4.70	0.00
2	2.03	1.04	0.00	35.10	1.62	15.27	44.93	0.00
3	3.24	7.07	0.32	44.31	0.00	1.05	34.34	9.69

**Table 4:** Elemental composition (weight percentage) of the internal part of the service exposed sample obtained by EDX analysis at two spots on the material’s surface (the places marked 1–2 in Figure 9).

No.	C (%)	Si (%)	V (%)	Cr (%)	Mn (%)	Fe (%)	Ni (%)	Nb (%)
1	2.73	28.62	34.80	12.92	0.09	5.36	15.44	0.00
2	2.42	0.00	0.66	32.53	0.00	15.86	48.22	0.32

**Table 5:** Elemental composition (weight percentage) of the external part of the service exposed sample obtained by EDX analysis at the spot on the material’s surface (the place marked 1 in Figure 11).

No.	C (%)	Si (%)	V (%)	Cr (%)	Mn (%)	Fe (%)	Ni (%)	Nb (%)
1	4.73	7.42	0.48	34.14	0.00	2.07	5.77	45.39

**Table 6:** Hydrogen and methane content in investigated alloy samples.

Alloy Sample	Hydrogen Content (ppm)	Methane Content (ppm)
service exposed	77.1	134.3
reference	32.8	40.0

**Table 7:** Basic mechanical parameters of the investigated alloy samples.

Alloy Sample	σ for 0.2% Plastic Deformation (MPa)	Young’s Modulus (GPa)	σ_max_ (MPa)	ε for σ_max_ (%)
service exposed	356	150	491	1.8
reference	323	163	559	6.3

## Data Availability

The data presented in this study are available on request from the corresponding author. The data are not publicly available due to ongoing further studies in the field.
